# Burn Injuries Resulting from Hot Water Bottle Use: A Retrospective Review of Cases Presenting to a Regional Burns Unit in the United Kingdom

**DOI:** 10.1155/2013/736368

**Published:** 2013-12-22

**Authors:** Shehab Jabir, Quentin Frew, Naguib El-Muttardi, Peter Dziewulski

**Affiliations:** ^1^Burn Service, St. Andrews Centre for Plastic Surgery and Burns, Chelmsford, Essex CM1 7ET, UK; ^2^Burn Research Group, St. Andrew's Anglia Ruskin (STAAR) Research Unit, Postgraduate Medical Institute, Anglia Ruskin University, Essex CM1 1SQ, UK

## Abstract

*Introduction.* Hot water bottles are commonly used to relieve pain and for warmth during the colder months of the year. However, they pose a risk of serious burn injuries. The aim of this study is to retrospectively review all burn injuries caused by hot water bottles presenting to our regional burns unit. *Methods*. Patients with burns injuries resulting from hot water bottle use were identified from our burns database between the periods of January 2004 and March 2013 and their cases notes reviewed retrospectively. *Results*. Identified cases involved 39 children (aged 17 years or younger) and 46 adults (aged 18 years or older). The majority of burns were scald injuries. The mean %TBSA was 3.07% (SD ± 3.40). Seven patients (8.24%) required debridement and skin grafting while 3 (3.60%) required debridement and application of Biobrane. One patient (1.18%) required local flap reconstruction. Spontaneous rupture accounted for 48.20% of injuries while accidental spilling and contact accounted for 33% and 18.80% of injuries, respectively. The mean time to heal was 28.87 days (SD ± 21.60). *Conclusions*. This study highlights the typical distribution of hot water bottle burns and the high rate of spontaneous rupture of hot water bottles, which have the potential for significant burn injuries.

## 1. Introduction

Hot water bottles are commonly used within the United Kingdom to provide warmth during the colder seasons and to relieve pain associated with conditions such as pancreatitis, cholecystitis, back pain, and pain related to menstruation. There are no formal figures on the number of hot water bottles sold within the United Kingdom; however, in Australia it is believed that well over 500,000 bottles are sold annually [1]. Hence it would not be unreasonable to assume this number to be well above a million bottles a year within the United Kingdom where the population is almost three times greater and the climate even colder. The financial hardships following the United Sates financial crisis of 2007-2008 resulted in a global recession with an immediate knock-on effect in the European region with the United Kingdom also being significantly affected [2]. A number of causes for the financial crisis have been postulated, each with different degrees of importance, and include high risk financial products, undisclosed conflicts of interest, and failure of the regulators of the financial industry [2]. The crisis resulted in the loss of thousands of jobs and a decrease, or even stagnation of pay, fostering an environment of austerity within the home. This, together with recent increases in energy prices, means that the general public are increasingly opting for alternative methods of keeping warm which has resulted in an increased demand for and usage of hot water bottles within the United Kingdom [3, 4].

Furthermore, heat is a well recognised nonpharmaceutical agent for the relief of pain among the general population and studies have provided evidence of its efficacy [5]. According to the gate control theory of pain, tactile stimuli (whether they are heat or light pressure) may help to decrease the perception of pain within the brain by stimulation of large diameter fibres. This results in closure of the “pain gate” in the spinal cord thus inhibiting the transmission of these impulses to the brain and their perception as a painful sensation within the brain [6]. Erythema ab igne is the term used to describe erythematous reticulated hypermelanotic lesions on the skin caused by repeated exposure to low level heat over the same location. It was common when households used wood-burning stoves for heat but declined in prevalence with the introduction of central heating [7]. The commonest contemporary cause of erythema ab igne is the application of a hot water bottle to the skin surface, although recently other causes such as electric heating pads and laptops have also been implicated [8]. The exact pathophysiological mechanisms underlying its development are unclear although epidermal atrophy, vasodilation, and dermal deposition of melanin and haemosiderin have all been implicated [7]. Treatment of erythema ab igne usually involves removal of the heat source. Continued exposure may lead to permanent dyspigmentation and other longer term sequelae include squamous cell carcinoma and Merkel cell carcinoma [7]. Erythema ab igne is well described in the literature and most members of the general public are aware of it [9, 10]. However, a much more serious consequence, and one which is less well described in the literature and less well appreciated by the general public, is the possibility of burn injuries following the use of hot water bottles.

The aim of this study is to retrospectively review all burn injuries caused by hot water bottles presenting to St. Andrews Centre for Plastic Surgery and Burns, a tertiary referral centre for burn injuries located in the East of England. This would help us determine the mechanisms of burn injuries via hot water bottles and provide information regarding the morbidity and mortality of these injuries. It is hoped that this information would then inform an educational campaign to ensure safer use of hot water bottles among the British public as well as in countries outside the United Kingdom.

## 2. Methods

St. Andrews Centre for Plastic Surgery and Burns has been managing burn patients of all ages and of all severities for the past 27 years [11]. It consists of an 8-bed burns intensive care unit (BICU), adult's and paediatric burn rehabilitation wards, and a dedicated burns outpatient department. It deals with between 850 and 1000 burn admissions per annum, in addition to approximately another 900–1000 patients being treated on an outpatient basis. The vast majority of admissions are secondary to scald and flame burns (80–85%) with chemical burns and electrical burns contributing to around 8–10% and 3–5%, respectively.

Patients with burns injuries resulting from hot water bottle use were identified from our burns database between the periods of January 2004 and March 2013. Institutional review board approval was not required due to the retrospective nature of the study involving review of patient notes. Case notes of identified patients were then retrieved and reviewed. In terms of data extraction, specific emphasis was placed on total burn surface area (%TBSA) involved, the mechanism of injury and outcomes such as time to healing, need for surgery and need for intensive care unit (ICU) admission.

## 3. Results

A total of 85 burn injuries caused by hot water bottles were identified from our database.  Table 1 provides burn injury demographics.

### 3.1. Date of Injury

**Figure 1:**
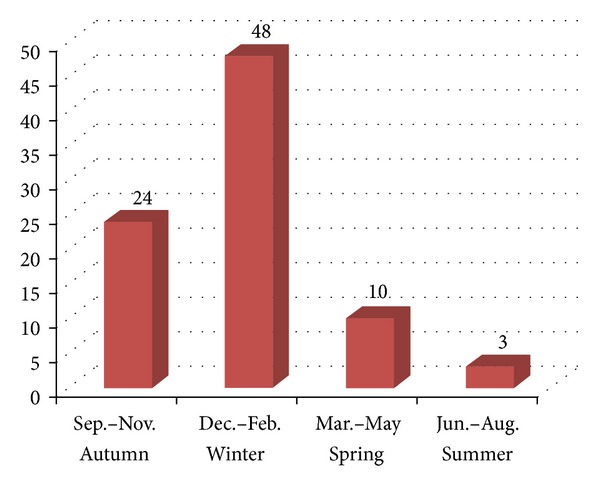
Breakdown of number of hot water bottle burns by season.

48 injuries occurred between the winter months of December and February while only 3 burn injuries occurred between the summer months of June and August.  Figure 1 provides a breakdown of the number of injuries occurring during the different seasons of the year.

### 3.2. Delay in Presentation

There were a total of 7 delayed presentations with the rest of the injuries presenting either on the day of or the day after injury. The average length of delay for all 7 cases was 8.8 days. In 2 cases there were extensive delays in presentation with one patient presenting at 3 weeks and another presenting at 2 months to our department. In both these cases the injury was conservatively managed by the patient's local GP. Both patients were eventually referred to our unit due to problems with healing. Apart from these cases, the remaining 5 cases presented between 5 and 10 days after injury.

### 3.3. Mechanism of Injury

**Figure 2:**
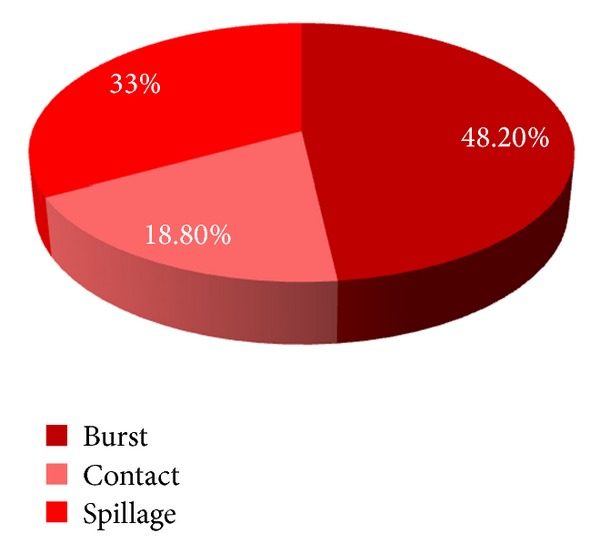
The relative contribution of each mechanism to burn injury.

Three mechanisms of injury were noted and included contact injuries, accidental spillage, and hot water bottle burst injuries. A more detailed definition of each of these three mechanisms is provided in Table 2. The relative contribution of each mechanism is summarised in Figure 2 while the mean %TBSA for each mechanism is provided in Table 3. The relative contribution of each mechanism for injuries in male patients and female patients is provided in Table 4.

### 3.4. Past Medical History

There was no significant past medical history in 64 cases. Out of the remaining 21 cases, 9 patients had a significant neurological disorder such as, paraplegia, Friedrich's ataxia, spina bifida, or nerve injury. Another 8 patients had a history of conditions causing chronic pain (back pain, chronic pancreatitis, arthritis) and 4 patients had a history of diabetes.

### 3.5. Social History

In 24 cases the social history was not recorded. In 51 cases the social history recorded is not relevant to the purposes of this study. However in the remaining 10 cases there were factors within the social history that suggested hot water bottle use was related to financial difficulties. In 3 of these 10 cases, the patients sited financial hardship as a direct reason for hot water bottle use.

### 3.6. Management and the Need for Surgery

**Figure 3:**
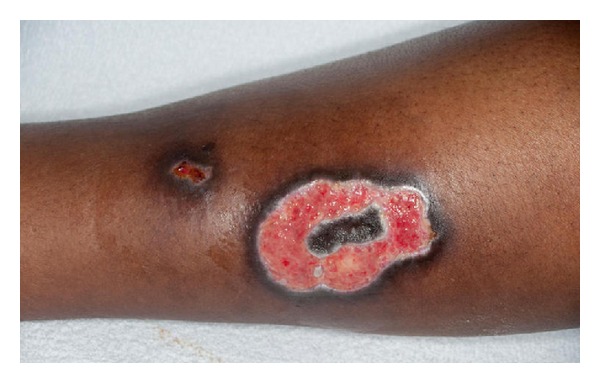
A full thickness burn to the shin sustained via prolonged contact with a hot water bottle. This patient required split-skin grafting to aid healing.

In 73 patients the burn injuries were managed conservatively via nonadherent dressings. Seven (8.24%) patients required debridement and split-skin grafting (see Figure 3) with a further 3 (3.5%) patients undergoing debridement and application of Biobrane (a biosynthetic skin substitute consisting of nylon fibres embedded in silicone to which collagen has been chemically bound). One patient with a mixed thickness burn involving a TBSA of 20.5% (19.5% partial thickness and 1% full thickness) required wound debridement via Versajet and application of Biobrane. This patient had two visits to theatre, one for the above mentioned procedure and a second one for removal of Biobrane. Another patient sustained a full-thickness burn to the right hand comprising a TBSA of approximately 0.3% which required debridement and application of a full thickness skin graft. However, the graft failed to take and the patient required a pedicled flap for reconstruction of the residual defect.

### 3.7. Wound Infection and Antibiotic Usage

A total of 43 patients had positive swab results (55.6%). The most common organisms cultured included *Staphylococcus aureus*, *Pseudomonas aeruginosa*, *Escherichia coli,* and beta-haemolytic *Streptococcus*. Antibiotics were prescribed in cases where there were clinical signs of infection with a positive swab result or in situations where there was a presumed high probability of subsequent infection. The most commonly prescribed agent was co-amoxiclav which has a broad-spectrum of antibacterial activity.

### 3.8. Admission to Burns Intensive Treatment Unit (ITU) and Mortality

None of the patients in our series required admission to our burns ITU and we did not have any mortalities as a result of burns sustained via hot water bottle use.

### 3.9. Reason for Hot Water Bottle Use

The reason for hot water bottle use was not recorded by the history taker in 68 cases. In the remaining 17 cases, 10 patients used hot water bottles to relieve pain. These patients has chronic painful conditions such as sickle cell crises, abdominal pain (due to chronic pancreatitis in 1 case), period pain, back pain, and pain due to ovarian cysts. The remaining 7 patients used hot water bottles to warm themselves up as they were feeling cold. Hence it appears that the primary usage of hot water bottles is to relieve pain in almost any part of the body and to help people warm up in a cold environment.

### 3.10. Site of Injury

**Figure 4:**
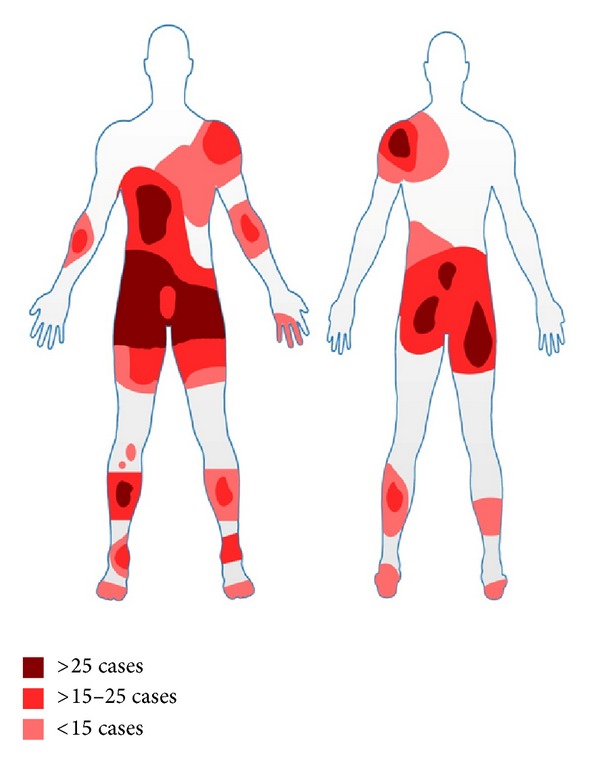
Body map showing areas of the body most commonly injured with decreasing frequency.

The commonest sites of injury are provided in the form of a body map in Figure 4.

## 4. Discussion

There is a paucity of studies looking at the epidemiology of burns caused by hot water bottles in comparison to other devices used for a similar purpose such as electric heating pads and electric blankets [12–16]. A literature review performed on Medline and Google Scholar for burns caused by hot water bottles revealed two studies, one from China (Ben et al.) and another from Australia (Whittam et al.) [1, 17]. Apart from this there are a number of case reports involving burns caused by hot water bottles: a perianal burn sustained following use of a hot water bottle to relieve a painful perianal fissure [18], partial thickness burns resulting from loss of sensory and nociceptive feedback following breast reconstruction surgery [19, 20], and 2 cases of foot burns in patients with diabetic neuropathy [21].

This is the first study, within Europe, of the epidemiology of burns caused by hot water bottles. Ben et al. assert that hot water bottle use within developed countries has greatly declined and that one would be hard-pressed to find them being used here. However, this study and the Australian study confirm that they are still being used in significant numbers within developed countries. Annually estimated 500,000 hot water bottles are sold within Australia [1]. No such statistics are available for the UK, but one can safely assume that the number will be significantly higher in the UK given the much larger population within the UK, the much colder weather encountered and their popularity within UK households.

**Figure 5:**
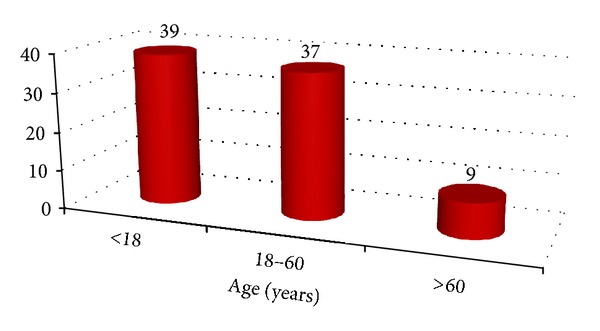
Relationship between burn injuries and age.

With regard to age distribution of burns, children and the elderly appear to comprise the majority of injuries (see Figure 5). This is not surprising as they are more vulnerable to this type of injury. Furthermore, women appear to be at a higher risk of injury than men because they seem to be fonder of using a hot water bottle than men. This result correlates with that of Ben et al. and Whittam et al. where again greater numbers of women were found to use hot water bottles and hence more likely to sustain burn injuries compared to their male counterparts.

The vast majority of injuries (72 injuries in total) appear to occur between autumn and winter (between September and February) with a sharp decline in spring and summer (13 injuries in total). This is most likely secondary to the general public seeking alternative modes of keeping warm beyond that of the more expensive traditional gas and electric central heating. It has also been shown that repetitive cold stimulation results in an aching pain sensation which eventually radiates beyond the site of stimulation [22]. Thus, patients suffering from painful conditions may experience an exacerbation of their pain symptoms when the ambient temperature drops resulting in them using a hot water bottle to relieve their symptoms as was the case with certain patients in our study.

Apart from a few cases, almost all patients presented immediately to their local hospital soon after injury. This was more common in injuries where there was a direct splash of hot water onto the skin via either accidental spilling or bursting of the hot water bottles, compared to burn injuries via contact with hot water bottles. Contact injuries present more insidiously as in most circumstances the patient losses consciousness, most commonly falling asleep, or have some degree of sensory deficit in the region of the body that the bottle is in contact with. This results in a reduced ability to recognise and respond to painful stimuli. Hence, these patients are less likely to recognise burn injuries until they have caused a significant degree of injury. In our study there were 3 cases of pure full-thickness burns with an average TBSA of 0.43%, all of them caused by prolonged contact with a hot water bottle due to sensory deficits.

The mean %TBSA of this study is similar to that of Ben et al., being in the range of approximately 3%. It appears that hot water bottles have the capacity to cause a reasonable sized burn in their users. The size of the burn is greater if the injury is sustained via a burst mechanism with spill/leak mechanisms being the next most serious. Contact burns on other hand appear to cause a smaller area of damage but with deeper tissue involvement.

The abdomen, chest, thighs, buttocks, perineum, lower legs, and feet are the most common site to be affected by burns injuries (see Figure 4). This appears to be due to most patients feeling colder (such as at the lower legs and feet) or experiencing pain (such as at the abdomen) in these regions of the body and hence placing the bottle at these positions.

In this study we also assessed the time taken for complete healing to occur, a parameter which none of the previous studies considered. The mean time to heal in children was 24.06 days, while in adults it was 32.34 days, with the overall mean being 28.19 days. It has been shown that wounds in children heal quicker and this is further attested to by our study [23]. In addition, it also enables us to appreciate the significant time span necessary for such an injury to heal. Frequent review and dressing changes are necessary and this may have implications for the patient and the National Health Service in terms of cost and time spent having these injuries treated.

None of the patients in our study required admission to our burns ITU. In addition there were no fatalities in our study. In the Chinese study 2 deaths were reported, while in the Australian study 1 death was reported. In both studies the deaths were in elderly patients with multiple other comorbidities such as congestive cardiac failure, diabetes, ischaemic heart disease, and pneumonia.

There was only one case of severe infection after burn in our study. This patient had a 3-day delay prior to presentation. Wound swabs showed a heavy growth of unidentified coliforms which were sensitive to co-amoxiclav. Following debridement, spilt skin grafting failed with the patient requiring reconstruction eventually with a pedicled reverse flow homodigital flap.

It is apparent that both patient misuse and poor manufacturing quality of hot water bottles may contribute to burn injuries. Hence, both of these factors may need to be addressed in order to reduce the number of burn injuries via hot water bottles. The public needs to be made aware of the risks of hot water bottle use and techniques which may be followed which may reduce the risk of burn injury from them. Following the mechanisms of injury identified in this study we recommend the methods discussed below to reduce the risk of burn injuries from hot water bottles (note that certain methods may help to prevent injury by more than one mechanism).

To prevent burst injuries the following recommendations are provided:

- frequently examining the bottle for any signs of wear and tear,
- making sure that any bottles purchased have been tested to BS1970:2006 standards,
- expelling all air above the water level before sealing with care taken to prevent injury from the escaping hot steam,
- discarding the bottle 2 years after initial purchase for a new bottle.

To prevent spill injuries the following recommendations are provided:

- making sure the stopper is securely screwed on,
- not filling the bottle to its brim but to about three-quarters full,
- filling the bottle with hot water and not boiling water.

To prevent contact injuries the following recommendations are provided:

- wrapping the bottle in a towel to prevent direct contact,
- abstaining from taking the bottle to bed.

Other recommendations include the following:

- care when used by those with sensory deficits as these individuals are at risk of a more serious thermal injury due to the loss of nociceptive feedback and impaired thermoregulatory capacity,
- care when used by the elderly with multiple co-morbidities as it may involve a prolonged course of recovery,
- care when used by children who are also a particularly vulnerable group,
- storage in a cool dry place where the bottle is not in contact with chemical agents which may affect the integrity of the material that the bottle is made of.

Following first aid, other factors such as the need for fluid resuscitation, ITU admission, debridement, and grafting are dependent on the %TBSA and the depth of the burn injury. What is clear from this study is that the vast majority of burn injuries caused by hot water bottles may be managed conservatively; however a small number may require application of split-skin grafts to aid healing.

With reference to tackling the issue of poorly manufactured bottles, serious thought may need to also be given to the current quality criteria, namely, the BS1970:2006 standards which are the benchmark criteria for hot water bottle manufacture. A recent study into rupture of a hot water bottle 3 months into its use by a rubber and plastics consultancy firm concluded that the BS1970 criteria may need to be revised as they were not sufficiently rigorous or demanding [24, 25].

Our burns unit has organised an awareness campaign in partnership with a local university to disseminate the results of this study. A preliminary report of the study findings resulted in widespread media coverage of the issue at both national and international level [26]. As the age-old adage goes, “prevention is better than cure”, hence in situations where one feels cold it is advisable to reduce loss of heat, by wrapping up in blankets, for example, rather than applying extrinsic heat to warm oneself up and to use painkillers for pain rather than expectant measures such as a hot water bottles [27]. We hope this study will go a long way towards educating healthcare staff and members of the general public about the possible dangers of hot water bottle use and what could be done to reduce the likelihood of burn injuries from them.

## Figures and Tables

**Table 1:** Essential burn demographics.

Age	25.7 years (9 days to 76 years)

Gender (male : female)	38 : 47 (44.7% : 55.3%)

%TBSA	3.07% (0.10–25.50%)

Adults (18 years or older) : children (17 years or younger)	46 : 39

Length of stay	1.29 days (0–28 days)

Time to heal (days)	28.19 days (6–109 days)

Ethnicity	Caucasian or British Caucasian—49
	Mixed—0
	Asian or Asian British—18
	Afro-Caribbean or Afro-Caribbean British—15
	Arab or British Arab—3

Operations	Debridement and spilt skin grafting—7
	Debridement and application of Biobrane—3
	Pedicled flap—1

Inhalational injury	0

**Table 2:** Detailed definition of injury mechanism.

Mechanism	Definition
Contact injury	Burn injury caused by the touching or meeting of the surface of an excessively hot hot water bottle with the skin

Accidental spillage	A burn injury caused by the accidental spillage of boiling water from the designated opening of the hot water bottle used for filling the bottle

Bottle burst injury	Sudden, unexpected breakage of the hot water bottle either spontaneously or due to patient misuse leading to release of boiling water and causing a burn injury

**Table 3:** Mean %TBSA caused by each mechanism.

Mechanism of injury	Mean % TBSA
Contact	0.89% (0.10–7%)
Spill	2.65% (0.30–10%)
Burst	3.94% (0.25–20.50%)

**Table 4:** Relative contribution of each mechanism for injuries in male patients and female patients.

Female (total no. 47)	Male (total no. 38)
Burst—24 (51%)	Burst—17 (44.7%)
Spill—15 (31.9%)	Spill—14 (36.9%)
Contact—8 (17.1%)	Contact—7 (18.4%)

## References

[1] Whittam A, Wilson A, Greenwood JE (2010). Burn wounds caused by hot water bottles: audit and loop closure. *Eplasty*.

[2] http://www.nber.org/papers/w14631.pdf.

[3] http://www.bbc.co.uk/news/business-24562930.

[4] http://www.dailyfinance.com/2011/02/02/hot-water-bottles-and-other-old-fashioned-ways-to-keep-warm-on-t/.

[5] Nadler SF, Steiner DJ, Erasala GN, Hengehold DA, Abeln SB, Weingand KW (2003). Continuous low-level heatwrap therapy for treating acute nonspecific low back pain. *Archives of Physical Medicine and Rehabilitation*.

[6] Melzack R, Wall PD (1965). Pain mechanisms: a new theory. *Science*.

[7] Steadmon MJ, Riley KN (2013). Erythema Ab Igne: a comeback story. *Journal of Pediatrics*.

[8] Miller K, Hunt R, Chu J, Meehan S, Stein J (2011). Erythema ab igne. *Dermatology Online Journal*.

[9] Chandramohan K, Bhagwat P, Arun T, Mohan S (2011). Erythema AB igne of chest in a patient with pulmonary tuberculosis. *Indian Journal of Dermatology*.

[10] Poustinchian BR, Pohlman DJ (2012). Erythema ab igne. *Journal of the American Osteopathic Association*.

[11] Roberts G, Lloyd M, Parker M (2012). The Baux score is dead. Long live the Baux score: a 27-year retrospective cohort study of mortality at a regional burns service. *The Journal of Trauma and Acute Care Surgery*.

[12] Diller KR (1991). Analysis of burns caused by long-term exposure to a heating pad. *Journal of Burn Care and Rehabilitation*.

[13] Stevenson TR, Hammond DC, Keip D, Argenta LC (1985). Heating pad burns in anesthetic skin. *Annals of Plastic Surgery*.

[14] Ozgenel E, Ozcan M (2003). Heating-pad burn as a complication of abdominoplasty. *British Journal of Plastic Surgery*.

[15] Gosselin TK (2003). Thermal wounds following heating pad use. *Clinical Journal of Oncology Nursing*.

[16] Williams E (1962). Misuse of electric blankets. *British Medical Journal*.

[17] Ben DF, Chen Xu L, Xia ZF (2004). Hot-water bottle burns: a review of 294 cases treated in Changhai Hospital Burn Centre in the period 1991–2001. *Annals of Burns and Fire Disasters*.

[18] Lapid O, Walfisch S (1999). Perianal and gluteal burns as a complication of hot water bottle treatment for anal fissure. *Burns*.

[19] Gowaily K, Ellabban MG, Iqbal A, Kat CC (2004). Hot water bottle burn to reconstructed breast. *Burns*.

[20] Jabir S, Frew Q, Griffiths M, Dziewulski P Burn injury to a reconstructed breast via a hot water bottle. *Journal of Plastic, Reconstructive and Aesthetic Surgery*.

[21] Jose RM, Vidyadharan R, Roy DK, Erdmann M (2005). Hot water bottles and diabetic patients: a cautionary tale. *British Journal of General Practice*.

[22] Mauderli AP, Vierck CJ, Cannon RL, Rodrigues A, Shen C (2003). Relationships between skin temperature and temporal summation of heat and cold pain. *Journal of Neurophysiology*.

[23] Viljanto J, Raekallio J (1976). Wound healing in children as assessed by the CELLSTIC method. *Journal of Pediatric Surgery*.

[24] Smithers Rapra Technology Limited Case study-hot water bottle. http://www.rapra.net/consultancy/case-studies-hot-water-bottle-premature-failure.asp.

[25] http://www.dailymail.co.uk/health/article-2233731/Number-scaldings-rise-Britons-choose-hot-water-bottles-turning-heating.html.

[26] http://www.itv.com/news/anglia/2012-11-20/new-research-highlights-hot-water-bottle-dangers/.

[27] London PS (1955). Hot water bottle burns. *British Medical Journal*.

